# Cost-effectiveness analysis of the nine-valent HPV vaccine in Italy

**DOI:** 10.1186/s12962-017-0073-8

**Published:** 2017-07-11

**Authors:** Francesco Saverio Mennini, Paolo Bonanni, Florence Bianic, Chiara de Waure, Gianluca Baio, Giacomo Plazzotta, Mathieu Uhart, Alessandro Rinaldi, Nathalie Largeron

**Affiliations:** 1 Faculty of Economics, Centre for Economic and International Studies (CEIS)-Economic Evaluation and HTA (EEHTA), University of Rome, Rome, Italy; 2 Institute for Leadership and Management in Health, Kingston University, London, UK; 3 Dept. of Health Sciences, University of Florence, Florence, Italy; 4 Mapi, Nanterre, France; 5 Institute of Public Health, Section of Hygiene, Catholic University of the Sacred Heart, Rome, Italy; 6 University College London, London, UK; 7 Mapi, Uxbridge, UK; 8 Sanofi Pasteur MSD, Lyon, France; 9 Sanofi Pasteur MSD, Rome, Italy

**Keywords:** Cost-effectiveness, Italy, HPV, Cervical cancer, Vaccination

## Abstract

**Background:**

In Italy HPV vaccination with the quadrivalent vaccine (Gardasil^®^) is offered actively and free of charge to girls aged 12 since 2007. A nine-valent vaccine (Gardasil 9^®^) received the European market authorization in 2015 to protect, with only 2 doses, against around 90% of all HPV positive cancers, over 80% of high-grade precancerous lesions and 90% of genital warts caused by HPV types 6/11.

**Methods:**

A dynamic transmission model simulating the natural history of HPV-infections was calibrated to the Italian setting and used to estimate costs and QALYs associated with vaccination strategies. The analyses compared two strategies with the nine-valent vaccine (cervical cancer screening and vaccination in girls only or vaccination in boys and girls) to four alternative strategies (cervical cancer screening and vaccination with quadrialent vaccine in girls only, in both boys and girls, with bivalent vaccine in girls and screening strategy only). The National Health Service perspective was considered.

**Conclusion:**

The switch to the nine-valent vaccine in Italy can further reduce the burden associated to cervical cancer and HPV-related diseases and is highly cost-effective.

**Results:**

Compared to the current vaccination program with quadrivalent vaccine, the nine-valent vaccine in a programme including girls and boys shows further reductions of 17% in the incidence of cervical cancer, 35 and 14% in anal cancer for males and females, as well as over a million cases of genital warts avoided after 100 years. The new technology is associated with an ICER of 10,463€ per QALY gained in universal vaccination, decreasing to 4483€ when considering the vaccine switch for girls-only.

**Electronic supplementary material:**

The online version of this article (doi:10.1186/s12962-017-0073-8) contains supplementary material, which is available to authorized users.

## Background

Human papillomavirus (HPV) [1] infection is the most common sexually transmitted infection and can be passed on through genital contact or by skin-to-skin contact [2, 3]. In the majority of cases, HPV infections are transient and cleared up within a few months after acquisition. However in some cases, HPV infection can persist and progress to cancer. The over 100 different HPV types identified have been divided into high and low risk types according to the risk for progression to cancer. High risk oncogenic HPV types (especially 16, 18, 31 and 45) are known to be primarily responsible for cancerous and precancerous lesions of the ano-genital area in both males and females. Overall, virtually all of cervical cancers, 88% of anal cancers, 43% of vulvar cancers, 70% of invasive vaginal carcinomas and 50% of all penile cancers worldwide can be attributed to HPV. The proportion of head and neck cancers caused by HPV is lower but not negligible [4]. Cervical cancer is the second most common cancer in young women in the European Union, and nearly all cases are attributable to HPV infection, the primary cause being the persistent infection of the genital tract by high-risk types (HPV 16/18/33/45) [5–7]. The second category, low-risk HPV types, do not cause cancer but are the agents of skin and genital warts. Low-risk HPV types include types 6 and 11, that are responsible for about 90% of all genital warts and can cause recurrent respiratory papillomatosis [8].

In Italy, a cervical cancer screening based on cytology is recommended every 3 years for women aged from 25 to 64 years [9, 10]. In addition to cervical cancer screening, HPV vaccination is offered actively and free of charge to girls aged 12 since 2007. HPV vaccines are designed to prevent HPV infection and HPV-related diseases, as demonstrated in clinical trials. There are currently two commercially available vaccines in Europe: the quadrivalent vaccine Gardasil^®^ and the bivalent vaccine Cervarix^®^. Both vaccines protect against high-risk HPV 16 and 18. The quadrivalent vaccine also includes protection against HPV 6 and 11, which are responsible for most cases of genital warts. The two vaccines are indicated for the protection of premalignant genital lesions (cervical, vulvar and vaginal) and cervical cancers; the indication of the quadrivalent vaccine also includes premalignant anal lesions, anal cancers and genital warts.

In Italy, each region is allowed to include additional age cohorts target group in the HPV vaccination programme; between 2014 and 2015, nine regions (Veneto, Liguria, Friuli-Venezia Giulia, Apulia, Sicily, Sicilia, Calabria, Molise, Trentino and Sardinia) extended the active vaccination programme to 12 year old boys. The vaccination program was initially based on a three dose regimen, but was subsequently changed to a 2-dose schedule in 2014.

A nine-valent vaccine (Gardasil 9^®^), developed with the aim to protect against nine HPV types (6, 11, 16, 18, 31, 33, 45, 52 and 58) was approved by the Food and Drug Administration in December 2014, and by the European Medicines Agency in June 2015 [11, 12]. Data confirm that types 16, 18, 31, 33, 45, 52 and 58 are amongst those most frequently detected [13]. Therefore, the nine-valent vaccine is expected to provide coverage against the majority of high-risk HPV types with carcinogenic properties [11]. With the addition of five HPV types compared to its predecessor, the nine-valent vaccine has the potential to prevent 70–90% of cervical, vulvar, vaginal and anal cancers, and 45–80% of precancerous cervical lesions [13, 14].

The total direct costs associated with the annual incident cases of HPV-related diseases [cervical cancers, precancerous cervical lesions, vaginal cancer, vulvar cancer, penile cancer, anal cancer, head and neck cancer, genital warts and recurrent respiratory papillomatosis (RRP)] including the cost of the diagnosis were estimated to be €529 million in 2011 in Italy [15]. The World Health Organization and the supervisory authority for public contracts in Italy, recommend that the decision-making process be based on both the quality of goods and services, as well as the best achievable price.

Several mathematical models have estimated the potential population impact of HPV vaccination on the burden of cervical cancer [16–22]. In Italy, like in other countries in Europe, there are numerous published studies assessing the cost-effectiveness of HPV vaccines [9, 19, 23–28]. Such studies conclude that the vaccination of girls with the bivalent and/or quadrivalent vaccines is cost-effective. More recently, the BEST II study estimated that universal vaccination (i.e. girls and boys vaccination) was also cost-effective in the Italian context [22]. On the other hand, the cost-effectiveness of 9-valent HPV vaccination was assessed in the United States. Three models were presented during the advisory committee on immunization practices (ACIP) meeting on February 26th 2015 [29–31]. Results of those studies showed that a universal vaccination programme with the nine-valent vaccine was likely to be cost-effective and even cost-saving compared to the current universal vaccination programme with the quadrivalent vaccine in the US, if a premium price of +10% was considered. Similar conclusions were drawn in Canada [32]. Brisson et al. [33] found that if the premium price of the nonavalent vaccine is below $13 per dose, the nine-valent vaccine would be cost saving with respect to the quadrivalent vaccine in the US setting. Robust cost-saving results were also found by Chesson et al. [34]. In Europe, the most recent study from Boiron et al. [35] showed that vaccinating 60% of girls and 40% of boys aged 9 in Austria with a nine-valent vaccine would substantially reduce the incidence of cervical cancer and be cost effective compared to the current strategy.

The objective of this study was to provide realistic estimates of the epidemiological and economic impact of the implementation of the 9-valent HPV vaccine program for both girls and boys in Italy compared to the current clinical practice using a 4-valent (HPV 6/11/16/18) or bivalent (HPV 16/18) vaccine for girls only, in a short and long-term horizon from the national health service perspective.

## Methods

### Mathematical model

A previously published US model, simulating the natural history of HPV-infections and estimating the cost associated with HPV-related diseases, has been extended to account for infections and diseases attributable to HPV genotypes 31, 33, 45, 52, 58 and adapted to Italy in order to estimate the cost-effectiveness of the nine-valent vaccine [19, 20].

The model is a deterministic, dynamic, ODE-based susceptible-infected-recovered-susceptible (SIRS) transmission model. This open-population based model consists of:

- A demographic model describing birth, ageing, and death.
- A behavioural model describing sexual mixing patterns.
- HPV infection and disease models describing transmission and disease occurrence.

Whereas HPV 6, 11, 16 and 18 are modelled separately, the five additional types are combined into a single set of compartments. All together, the model accounts for the transmission dynamics of nine HPV types: 16, 18, 6, 11, 31, 33, 45, 52, and 58, and simulate the occurrence of genital warts; RRP; precancers such as cervical intraepithelial neoplasia (CIN); cervical, vulvar, vaginal, penile, anal, and head/neck cancers related to these HPV types.

The current analysis follows a conservative approach and considers an additional clinical benefit of the nine-valent vaccine only for CIN, cervical and anal cancers. The status quo was assumed for other diseases.

In addition, as RRP, penile and head and neck cancers are not included in the vaccine label, those indications were not incorporated in the base case analysis, but they were considered in the sensitivity analyses.

Two different strategies with the nine-valent vaccine (cervical cancer screening and the nine-valent vaccine for girls only or both boys and girls) were compared to four alternative strategies:

- Cervical cancer screening and the quadrivalent vaccine for girls only;
- Cervical cancer screening and the quadrivalent vaccine for both boys and girls;
- Cervical cancer screening and bivalent vaccine for girls only;
- Screening strategy only.

### Epidemiological model parameters

The numerous model inputs of the epidemiological model are divided into demographics, sexual behavior, disease and treatment patterns, screening, and natural history of disease. The parameter estimates were derived from published data and a calibration process. Further details on the calibration material and techniques are presented in the section “Model calibration and validation”. The main set of epidemiological data are displayed in Additional file 1. All remaining model parameters and their values have been previously reported in detail in a technical report from Elbasha et al. [19].

#### Demographics

The model population reflects the current size and demographic characteristics (gender distribution and all-cause mortality rates) of the Italian population. These estimates were retrieved from the Italian National Institute of Statistics [10].

#### Sexual behaviour

Each age group consists of persons with low, medium or high sexual activity. Data on sexual behaviour in Italy were scarce or differed in several aspects from the inputs needed to inform the model. The results from the United Kingdom (UK) NATSAL-3 study were used. They were deemed to be applicable to the Italian setting according to experts’ opinion as well as being consistent with previously published economic literature (Haeussler et al.) [21, 22, 36].

The degree of sexual mixing among members of different age cohorts and sexual activity groups (0 representing no mixing, and 1 representing a maximal mixing) was set up in similar to the US model and adjusted during the calibration process [19].

#### Natural history of disease

The progression from infection to disease follows a similar natural history structure as the initial US model. Since transmission rates are not directly observable, calibration techniques were used to obtain the best set of parameters.

#### Disease and treatment patterns

Women with precancerous lesions [CIN, vaginal intraepithelial neoplasia (VaIN) and vulvar intraepithelial neoplasia (VIN)] or with cancer were classified into undetected, detected or treated categories. The proportion of women recognising their disease and seeking treatment and the proportion of treated women were estimated through the model calibration.

The women who had a benign hysterectomy and the ones who were treated and cured for cervical cancer were no longer at risk of cervical cancer. The incidence rates of hysterectomy by age were retrieved from the most recent Italian publication [9].

The annual probability of death for each HPV-related cancer, stratified by age and stage, were obtained by combining the data from two sources: the age-specific data from EUROCARE-5 and the stage-specific data from the BEST II study [22, 37].

#### Screening

In Italy, a cervical cancer screening with cytology is recommended every 3 years for women aged from 25 to 64 years [9, 10]. HPV testing as primary screening in women ≥30 years to be performed every 5 years has been introduced in a few regions, but data are still very scarce. Therefore, the focus of the analyses was Pap smear testing. The model reflected the current Italian situation where 77% of women receive gynecological cancer screening tests at least once every 3 years, as reported by the Italian national screening observatory (*Osservatorio nazionale screening*). The model was also informed by the age specific screening coverage rates in the past year, using two different sources to cover all the age groups [22, 38]. No screening for women aged 0–10 and 75+ was assumed.

The proportion of women who received a follow-up visit after an abnormal PAP smear test (70.52%) was estimated from the English Cervical screening programme 2013–2014, as no Italian source was found [39]. In terms of diagnostic performance, the sensitivity and specificity of the colposcopy were 90 and 48% respectively, whereas the specificity of the Pap test was 95.7% [9].

### Economic model parameters

The epidemiologic model adapted to the Italian population was then connected to the economic model. The latter simulates the costs and utilities associated with the HPV-cases and related diseases and assesses the impact of each prevention strategy. The inputs for the economic model are divided into vaccination strategy, vaccine properties, costs, and health-related quality of life.

#### Vaccination strategy

The current vaccination program with the bivalent and quadrivalent HPV vaccine in Italy is for girls at their 12th year of age, with recent data showing a coverage rate of 71.1% [40]. The vaccination consists of a two-dose schedule with a high adherence rate (proportion receiving the 2nd dose after the 1st one) of 90% [41]. It has been assumed that a vaccination programme with the nine-valent vaccine and or the addition of boys’ immunization would have the same performance (coverage and adherence).

#### Vaccine properties

The prophylactic efficacy of the vaccine or vaccine degree of protection was based on clinical trial data (Table 1) [42–47]. The duration of protection against vaccine types (6/11/16/18/31/33/45/52/58) was assumed to be lifelong. This parameter was tested in sensitivity analyses adopting a conservative approach with a lower duration of 20 years [19, 20]. The model makes a distinction between the level of protection against the infection and again the disease resulting from a breakthrough infection and considers different efficacy values for each. It is further assumed that these “breakthrough” infections are transmissible. The efficacy on head and neck, penile and RRP diseases was assumed to be conferred through protection against infection only. As they are not included in the vaccine label, these indications were not incorporated in the base case analysis but considered in the sensitivity analyses.

**Table 1:** Summary table on vaccine assumptions Giuliano et al. [44] for males and Elbasha and Dasbach [19] for females

Vaccine assumptions (proportion of exposed people avoiding the infection)	HPV 16	HPV 18	HPV 31, 33, 45, 52 and 58
Cervical cancer			
Vaccine efficacy for preventing cervical HPV16/18/31/33/45/52/58 infections			
Male*	0.411	0.621	0.411
Female**	0.76	0.963	0.76
Degree of protection of the vaccine against cervical HPV16/18 infections becoming persistent	0.988	0.984	0.988
Degree of protection of the vaccine against HPV16/18 -related CIN	0.979	1	0.979
Vaginal and vulvar cancers			
Vaccine efficacy for preventing vaginal/vulvar HPV16/18 infections			
Male*	0.411	0.621	
Female**	0.76	0.963	
Degree of protection of the vaccine against vaginal/vulvar HPV16/18 infections becoming persistent	0.988	0.984	
Degree of protection of the vaccine against HPV16/18-related/VaIN/VIN	1	1	
Anal cancers			
Vaccine efficacy for preventing anal HPV16/18 infections			
Male*	0.411	0.621	0.621
Female**	0.76	0.963	0.963
Degree of protection of the vaccine against anal HPV16/18 infections becoming persistent			
Male*	0.787	0.96	0.96
Female**	0.988	0.984	0.984
Degree of protection of the vaccine against HPV16/18 -related AIN	0	0	0
Penile and H&N cancers			
Vaccine efficacy for preventing anal/penile**/H&N HPV16/18 infections			
Male*	0.411	0.621	
Female**	0.76	0.963	
Degree of protection of the vaccine against anal/penile/H&N HPV16/18 infections becoming persistent			
Male*	0.787	0.96	
Female**	0.988	0.984	
Degree of protection of the vaccine against HPV16/18 -related AIN/PIN/H&N neoplasia	0	0	

* Preventing male genital infections through male vaccination is assumed to prevent transmission of genital infections to females

** Preventing female genital infections through vaccination is assumed to prevent transmission of genital infections to males

A two-dose regimen was considered in the model for the quadrivalent vaccine and also for the nine-valent vaccine to reflect the on-going trial. We assumed that if only one dose was administrated, the vaccine would have no efficacy.

In recent World Health Organization (WHO) guidelines on cervical cancer it was highlighted that the duration and strength of effectiveness of cross-protection was still to be demonstrated. Therefore, no cross-protection was assumed in the base case [48].

#### Cost of vaccination

In Italy, the vaccination programme is financed at Regional level and therefore it can be largely different in terms of age and number of the target cohorts, catch-up programmes and access procedure. For this reason, the cost of each vaccine dose is subject to a wide variability across the regions and over time [22]. The maximum price for public healthcare providers was set at €104.00, which corresponds to the ex-factory price per dose negotiated by the Italian agency for medicines. The cost of the 9-valent vaccine was not available in Italy since the product is not yet marketed. A theoretical base case price for the nine-valent vaccine of €120.00 was chosen, the equivalent of the price set in the public sector in the US.^1^ The price of both vaccines was varied in the sensitivity analyses. Moreover threshold analyses were conducted to find the cost-effective price for a ceiling ratio of 25,000–40,000€/QALYs, which is in line with Italian guidelines [49]. The administration cost was set at €6.6 [22].

#### Cost per episode of care

The costs per episode of care of each HPV-related disease, defined as the cost of management from diagnosis to resolution of the case, were estimated from the BEST II study and Baio et al. and are displayed in Table 2 [15, 22]. The productivity losses as a result of HPV disease were not included in the model.

**Table 2:** Summary table on costs and utilities for HPV-related disease

HPV-related disease	Cost (€)		References	Utility		References
	Females	Males		Males	Females	
CIN 1	452.0		[22]		0.8396	[22]
CIN 2	1485.0		[22]		0.7967	[22]
CIN 3, CIS	1971.8		[22]		0.8396	[22] (assumption on CIS)
Cervical cancer, local disease	20,652.7		[22]		0.54375	[22]
Cervical cancer, regional disease	35,930.4		[22]		0.5701	[22]
Cervical cancer, distant disease	34,574.7		[22]		0.4517	[22]
VaIN 2	3237.0		[22]		0.9793	[22]
VaIN 3, CIS	3237.0		[22]		0.9793	[22]
Vaginal cancer, local disease	7703.2		[22]		0.54375	[22]
Vaginal cancer, regional disease	19,835.9		[22]		0.5701	[22]
Vaginal cancer, distant disease	29,646.9		[22]		0.4517	[22]
Vulvar cancer, local disease	7183.7		[22]		0.54375	[22]
Vulvar cancer, regional disease	16,032.6		[22]		0.5701	[22]
Vulvar cancer, distant disease	20,365.4		[22]		0.4517	[22]
Penile cancer, local disease		10,497.6	[15]	0.7922		[22]
Penile cancer, regional disease		10,497.6	[15]	0.7922		[22]
Penile cancer, distant disease		10,497.6	[15]	0.7922		[22]
Anal cancer, local disease	9812.4	9812.4	[22]	0.653	0.69615	[22]
Anal cancer, regional disease	18,480.4	18,480.4	[22]	0.4175	0.5172	[22]
Anal cancer, distant disease	11,993.6	11,993.6	[22]	0.1998	0.2244	[22]
Head & Neck cancer, local disease	10,081.7	10,081.7	[22]	0.8171	0.7413	[22]
Head & Neck cancer, regional disease	28,572.1	28,572.1	[22]	0.5601	0.551	[22]
Head & Neck cancer, distant disease	28,572.1	28,572.1	[22]	0.5601	0.551	[22]
Genital warts	700.3	495.8	[15]	0.6961	0.7761	[22]
Recurrent respiratory papillomatosis	195,814.9	195,814.9	[15]	0.795698925	0.795698925	[53]

#### Cost of screening and diagnostic tests

The Italian reimbursement tariffs from the Italian Ministry of Health were used to extract the costs of PAP test, colposcopy and biopsy [50].

All costs were updated to 2014 Euros using the National Price Index for the whole community [51].

#### Health-related quality of life

Age-specific utilities for the Italian general population were not available in the literature. In a report from the *Istituto Superiore di Sanita’*, utilities for the Italian general population were assumed equal to the US population, thus the same assumption was used in the model [52]. Disease-related utilities were collected from the BEST II study [22] for all the diseases except for recurrent respiratory papillomatosis which was collected from Lindman et al. [53]. and are summarized in Table 2. Health utility values from the BEST II study were based on an Italian Time Trade-off study on HPV-related diseases [54]. We assumed that the quality of life for cancer survivors after successful treatment was the same as healthy women of the same age. The alternative set of utilities that were used for the sensitivity analysis is from Elbasha et al. [19, 20].

#### Discounting

Both costs and outcomes were discounted to present value at a rate of 3% per the Italian pharmacoeconomic guidelines [49]. Alternative discount rates of 1.5% for outcomes, 0 and 6% for both costs and outcomes were used in the sensitivity analysis.

### Model calibration and validation

Despite a good level of accuracy in the choice of the inputs, epidemiological results may not be consistent with observed Italian data reported in the national statistics. For this reason it was necessary to perform a calibration, the process in which epidemiological model inputs are tuned in order to obtain results closer to real data. The targets of the calibration are data on incidence and mortality rates of HPV-related diseases.

Overall and age-specific incidence data for cervical, anal, vaginal, vulvar and penile cancers were found in the report from the Institut Catala’ d’Oncologia (ICO)—Information center on HPV. The aim of this report was to compile and centralize updated data and statistics from the official Italian registry on HPV and related cancers; age specific values were extracted from the graphs [55]. The ITACAN database was used for incidence data on cancers in oral cavity, oropharynx and larynx. Overall incidence of genital warts was found in an Italian study (Baio et al.). This Italian study provided also overall incidence for CIN 1 and CIN 2–3 incidence. Nevertheless, the reported values were much lower than other available data (Hartwig). Therefore, UK data were also considered as a higher bound of plausible data.

Since the model studies HPV-induced diseases only, target epidemiological values were calculated by multiplying the incidence or the mortality with the percentage of the disease that can be attributed to HPV infection.

The proportion of diseases attributable to HPV infection (for the 2-valent, 4-valent or 9-valent vaccines) were found in the literature [13, 56]. The model was calibrated to attribute 73% of CIN 2/3 and cervical cancer incidence to HPV 16 and 18 and 17% to HPV 31, 33, 45, 52, and 58.

HPV-related head and neck cancers were calculated by weighting HPV-related oral cancers, oropharynx cancers and larynx cancers on their overall incidence.

The calibration process involved many rounds of iterations to move model outcomes closer to the targets. The following model outcomes were compared against the calibration target in each iteration: cervical cancer incidence, genital warts incidence, vaginal/vulvar/penile/anal/head and neck cancer incidence, and mortality rates of cervical/vaginal/vulvar/head and neck cancer.

The variables that affect all or most of the outputs are referred to as global variables. These include behavioural parameters, natural history of disease, transmission rates and all-cause mortality. They were first adjusted by changing transmission rates. The variables that affect only specific outputs are referred to as specific variables. These include disease-specific probability of death and rate of seeking treatment, and were used to fine-tune each disease area.

### Model analyses

The model was used to estimate the total number of disease events associated with HPV vaccine types (6/11/16/18/31/33/45/52/58-related); the incidence and mortality (cervical cancer, CIN, anal cancer and genital warts); the costs of vaccination, screening, diagnosis and management of the disease; the quality-adjusted life years (QALYs) of the model population. Results were reported over 100 years for the different strategies tested. Incremental cost-effectiveness ratios (ICERs) were then calculated by dividing the difference in the average accumulated costs by the average QALYs gained.

Sensitivity analyses were performed deterministically, modifying the value of one base case parameter at a time. The following key parameters were tested: vaccine price (with a low price of 56€ for the quadrivalent vaccine and Cervarix, and 80€ for the nine-valent vaccine), duration of protection (20 years), utilities from Elbasha et al. discount rates (1.5% instead of 3% for outcomes), and the inclusion of RRP, penile and head and neck cancer indications.

## Results

### Calibration

Most of the overall rates are very close to published data (less than 15% difference). Target were not matched closely for overall vaginal and vulvar cancer incidence and mortality. Age-specific values are generally difficult to calibrate, given the numerous parameters at stake and the diversity of the sources. Overall the calibrated age specific values were close to the observed data with the exception of the older age groups (75+).

### Epidemiological results

**Fig. 1:**
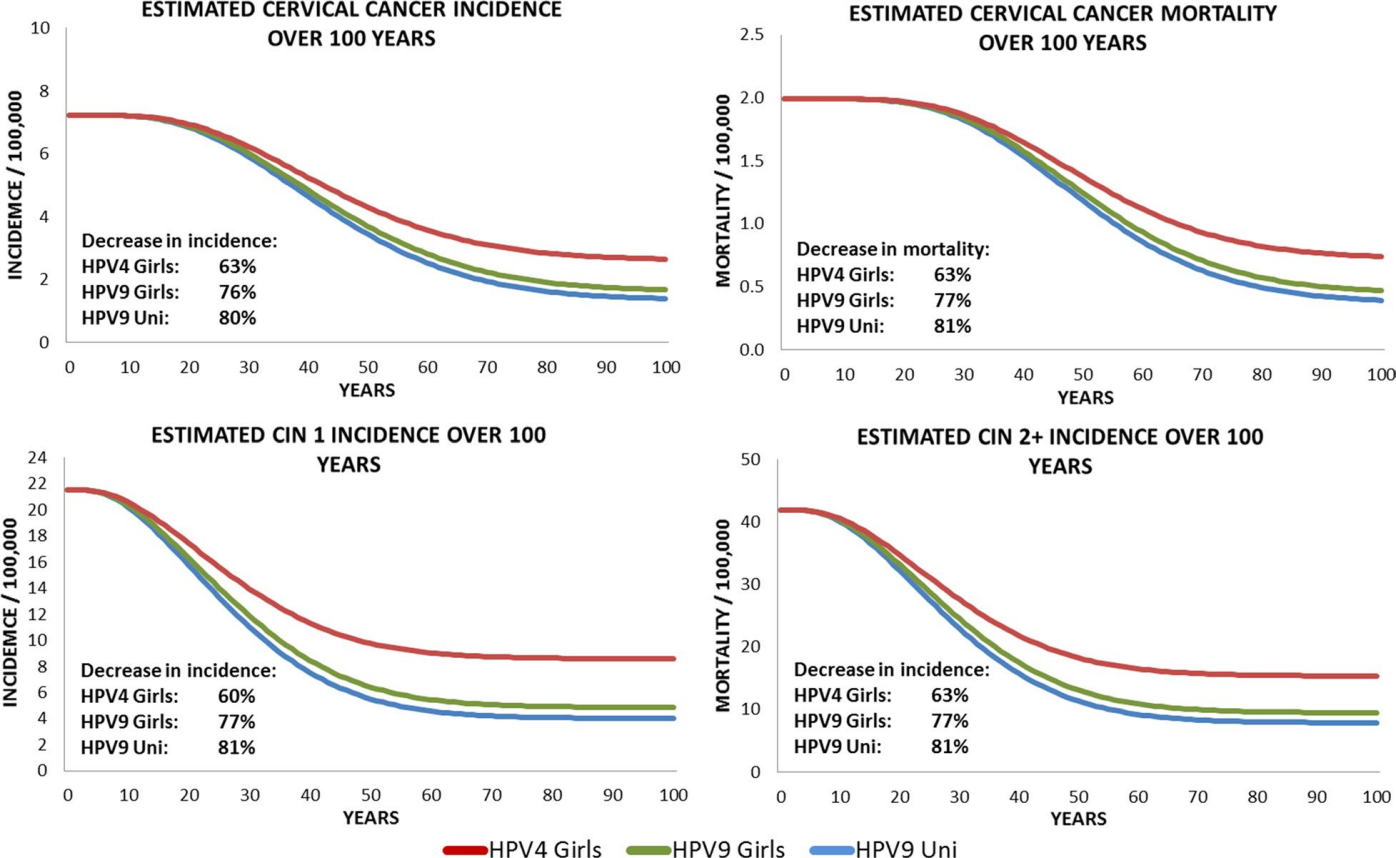
Epidemiological impact of three vaccination strategies on the incidence and mortality of cervical diseases (related to related to HPV 16/18/31/33/45/52/58)

The nine-valent vaccine girls-only vaccination and the quadrivalent vaccine girls-only vaccination are associated with a 76 and 63% decrease in incidence of cervical cancer respectively, over 100 years, as shown in Fig. 1. Overall, the estimated number of cervical disease events prevented with the nine-valent vaccine in 100 years in comparison with the quadrivalent vaccine was 16,678 for cervical cancer, 82,598 for CIN1, and 127,742 for CIN2+, as shown in Table 3. In total, the reduction in the number of cases of precancerous lesions (CIN 1, CIN 2/3) and genital warts occurred within 5th years of the start of the vaccination programme. The reduction in incidence of HPV-related cancers and deaths from HPV-related cancers was more gradual, reflecting the fact that HPV-related cancers are diseases with slower progression.

**Table 3:** Disease events prevented with the nine-valent vaccine universal vaccination in comparison with the current strategy (the quadrivalent vaccine girls-only vaccination)

Disease event	Years since start of vaccination programme			
	5	25	50	100
Females				
Cervical cancer	0	367	4623	22,640
CIN 1	23	6961	34,753	105,431
CIN 2/3	25	10,193	54,709	170,286
Vaginal cancer	0	0	13	88
VAIN 2/3	0	0	0	0
Vulvar cancer	0	1	21	130
Genital warts and HPV 6/11-related CIN 1	33	2438	6270	13,658
Genital warts	1411	61,285	161,443	358,140
Anal cancer	0	11	318	2619
Males				
Genital warts	6101	208,935	615,645	1508,705
Anal cancer	0	28	748	5492

**Fig. 2:**
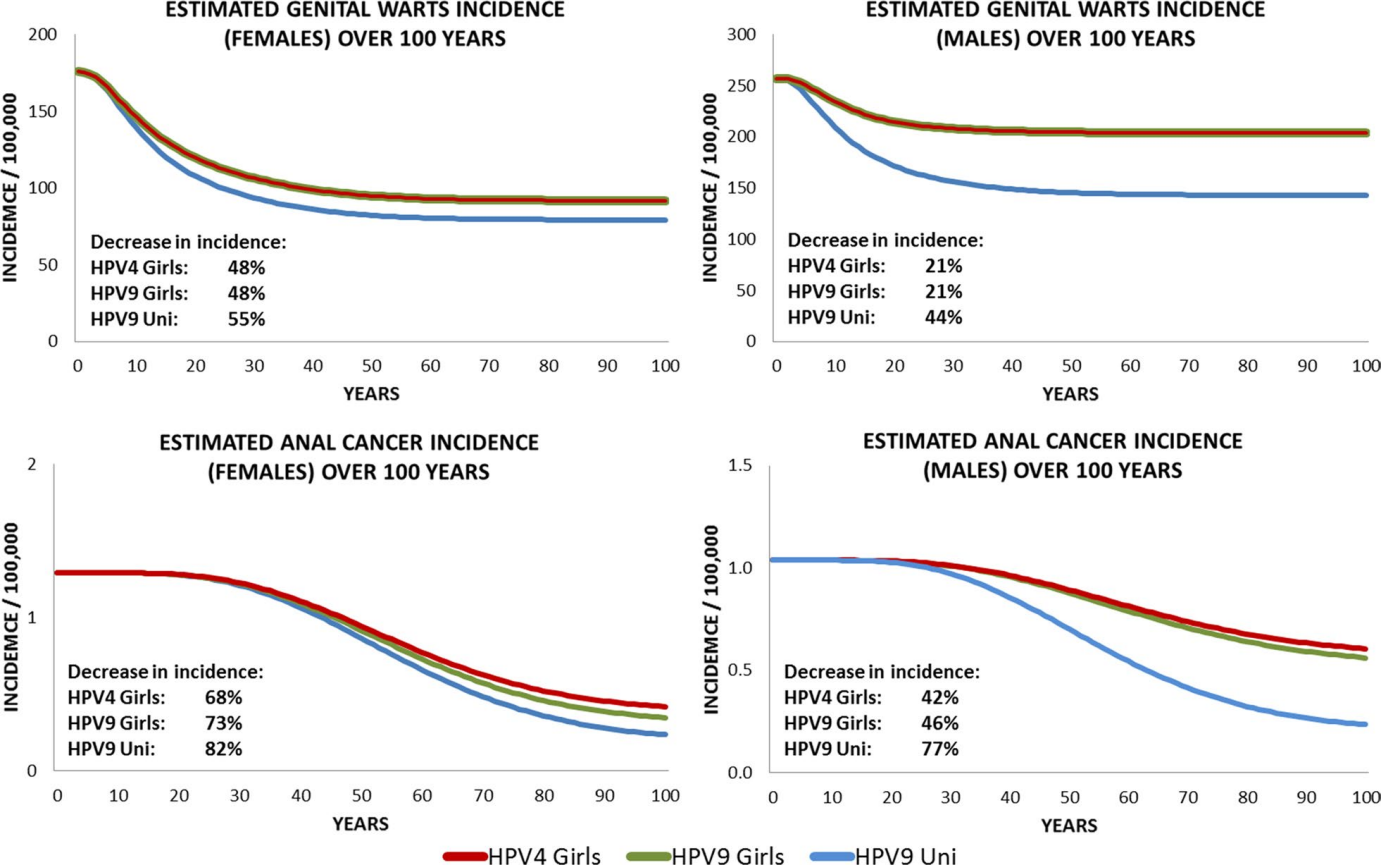
Epidemiological impact of three vaccination strategies on the incidence of genital warts and anal cancer (related to related to HPV 16/18/31/33/45/52/58)

Switching from a girl-only vaccination with the quadrivalent vaccine to a universal vaccination with the nine-valent vaccine showed significant health benefits. With respect to the quadrivalent vaccine, additional 22,640 prevented cases of cervical cancer, 105,431 of CIN1, and 170,286 of CIN2+ were associated with the universal vaccination. Furthermore, the comparison between the nine-valent vaccine universal vaccination and the quadrivalent vaccine girls-only vaccination (Table 3) estimated that vaccinating boys will prevent 1508,505 cases of genital warts among males, 358,140 cases of genital warts among females, and 8111 cases of anal cancer. This corresponds to an additional decrease in incidence with respect to the nine-valent vaccine girl-only vaccination of 4% in cervical cancer, 4% in CIN1, 4% in CIN2+ (Fig. 1), and 7% in genital warts among female (Fig. 2).

### Cost-effectiveness results

The ICER of the nine-valent vaccine with respect to the quadrivalent vaccine was 4483€/QALY when considering girls-only vaccination and 10,463€/QALY for a universal vaccination (Table 4). The implementation of the nine-valent vaccine universal vaccination in comparison to a girls-only program with the quadrivalent vaccine was associated with a cost per QALY gained of 13,541€. In the instance where the vaccination covered only girls, the nine-valent vaccine was cost-saving with respect to the bivalent vaccine.

**Table 4:** Cost effectiveness results of the base case analysis

Comparison		New technology		Comparator		Incremental costs	Incremental QALYs	Cost per QALY gained
New tech	Comparator	Costs	QALYs	Costs	QALYs			
HPV9 girls	HPV4 girls	€183.29	27.53857	€180.60	27.53797	2.69	0.0006	*€4483*
HPV9 girls	HPV2 girls	€183.29	27.53857	€188.92	27.53571	−5.63	0.00286	*Cost saving*
HPV9 universal	HPV4 universal	€213.64	27.54041	€206.63	27.53974	7.01	0.00067	*€10,463*
HPV9 universal	HPV4 girls	€213.64	27.54041	€180.60	27.53797	33.04	0.00244	*€13,541*

**Fig. 3:**
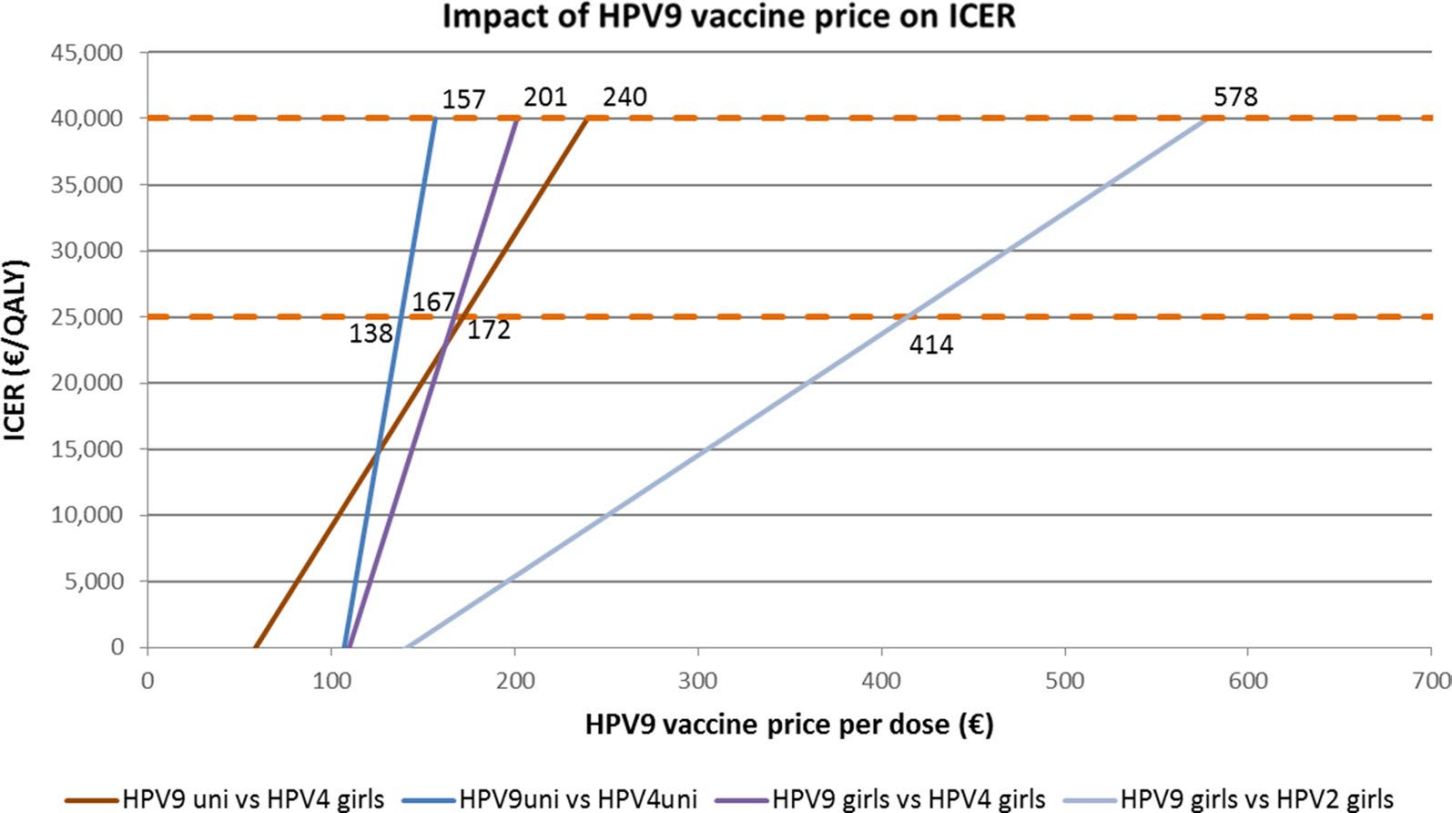
Price threshold analysis

The comparison of the nine-valent vaccine with the screening only strategy scored an ICER of 2592€/QALY for the girls only vaccination and 5855€/QALY in the case of a universal vaccination is selected. As shown in Fig. 3 switching from the quadrivalent vaccine to the nine-valent vaccine girls-only vaccination is cost-effective up to a price of €201 per dose, considering a threshold for the ICER of 40,000€/QALY. All the threshold prices can be found in Fig. 3.

### Sensitivity analyses

**Fig. 4:**
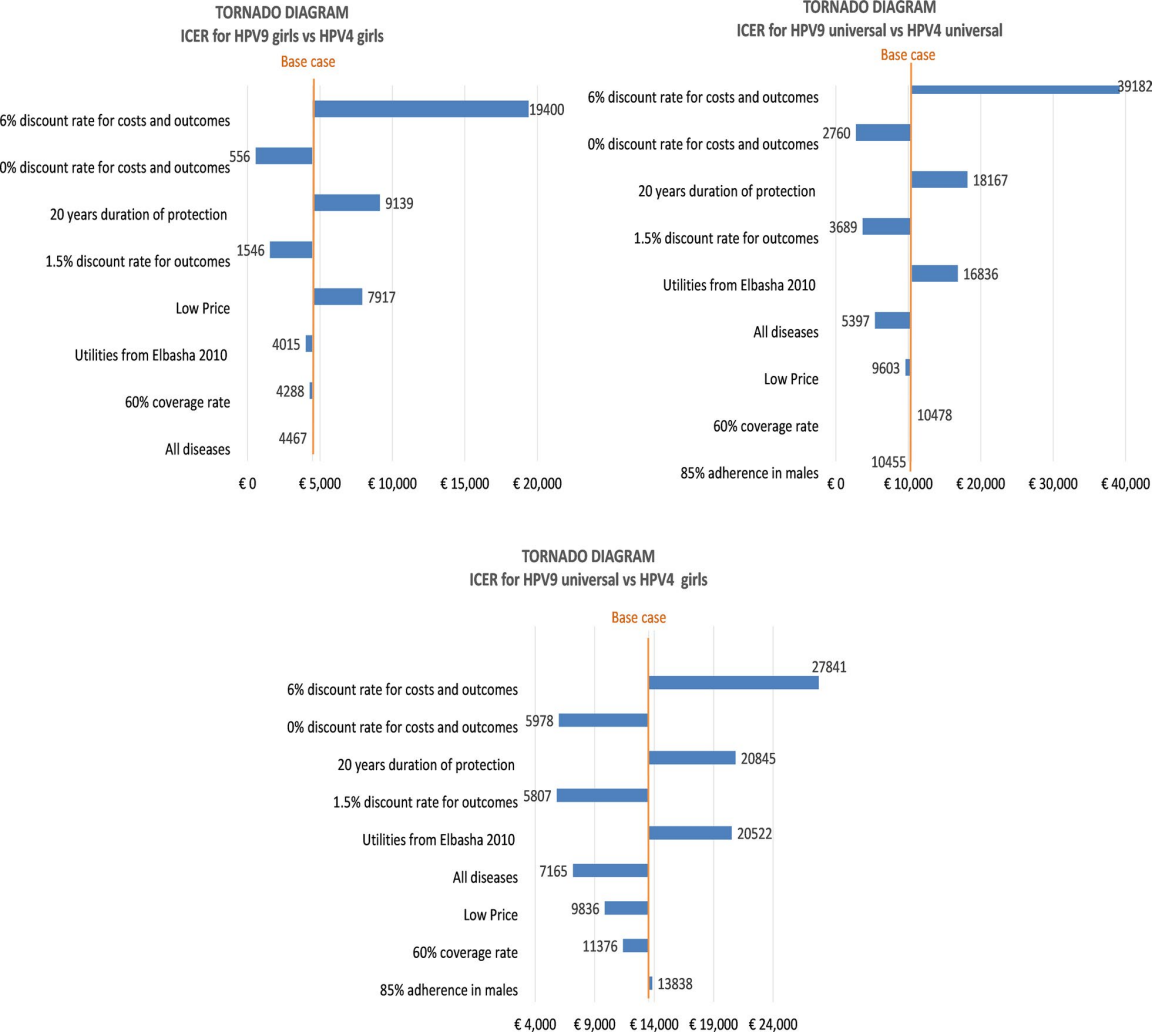
Tornado diagrams comparing the ICERs resulting from the sensitivity analysis

One-way sensitivity and scenario analyses were conducted and the cost-effectiveness results are displayed in Tornado diagrams in Fig. 4. The bivalent vaccine remained dominated by the nine-valent vaccine in all sensitivity analyses. The low price alternative decreased the ICER with respect to base case in the nine-valent vaccine universal vs the quadrivalent vaccine girls. In the other comparisons, considering that only the vaccine will change but not the target population, the ICER increased instead, but remained well beyond the minimum threshold of 25,000€/QALY. The ICERs were very sensitive to the discount rate. A higher discount rate of 6% substantially reduced both costs and outcomes and resulted in significantly higher ICERs for the three comparisons: 19,400€/QALY for the comparison between the nine-valent vaccine and the quadrivalent vaccine in a girls-only vaccination strategy, 39,182€/QALY for the nine-valent vaccine and the quadrivalent vaccine in a universal vaccination and 27,841€/QALY for the nine-valent vaccine universal strategy versus the quadrivalent vaccine girls-only strategy. Testing no discount rate greatly increased costs and outcomes and produced the lowest ICERs for the comparison between the nine-valent vaccine and the quadrivalent vaccine in a girls-only vaccination strategy (556€/QALY) and for the nine-valent vaccine and the quadrivalent vaccine in a universal vaccination (2760€/QALY). For the nine-valent vaccine universal strategy versus the quadrivalent vaccine girls-only strategy, the lowest ICER (5807€/QALY) was achieved while testing a lower discount rate of 1.5% only for outcomes and of 3% for costs. A 1.5% discount rate for outcomes-only also decreased significantly the ICER for the comparison between the nine-valent vaccine and the quadrivalent vaccine in a girls-only vaccination strategy (1546€/QALY) and the nine-valent vaccine and the quadrivalent vaccine in a universal vaccination (3689€/QALY). A decrease in the duration of protection from lifelong in the base case to 20 years resulted in higher incremental costs and lower incremental QALYs producing an overall ICER increase (9 139€/QALY for the comparison between the nine-valent vaccine and the quadrivalent vaccine in a girls-only vaccination strategy, 18 167€/QALY the comparison between nine-valent vaccine and the quadrivalent vaccine in a universal vaccination and 20 845€/QALY when comparing a universal vaccination with the nine-valent vaccine and a girls-only vaccination with the quadrivalent vaccine). The ICERs were not very sensitive to the lower coverage rates in girls-only; when considering the male vaccination in both strategies, a lower overall coverage rate of 60% decreased the ICER to 7165€/QALY. The change in utility values increased the ICER in the comparison with different target population, and remained close to the base case in the other two. Similarly, the inclusion of RRP, penile and H&N cancers indication left the ICER almost unchanged from the base case in the comparisons with the same target population, but it decreased the ICER to 7165€/QALY when comparing a universal vaccination with the nine-valent vaccine and a girls-only vaccination with the quadrivalent vaccine. A lower adherence rate in males (85%) resulted in an ICER almost unchanged in both comparisons considering universal vaccination.

## Discussion

In the current study, we assessed the cost-effectiveness of alternative vaccination strategies against HPV infection in the Italian setting comparing traditional bivalent and quadrivalent vaccines with the newly-available 9-valent vaccine.

The results show that the new nine-valent vaccine has a positive impact on cervical cancer and pre-cancerous lesions. This is of importance as the economic burden of cervical cancer in Italy is estimated at €147million [15]. The introduction of the new vaccination strategy with the nine-valent vaccine would reduce cervical cancer and CIN incidence by 77% in 100 years when vaccinating girls only, which is 13, 17 and 14% more for cervical cancer, CIN1 and CIN2+ respectively than with the quadrivalent vaccine girls-only vaccination strategy. Considering a girls-only vaccination, the analyses provide evidence that the nine-valent vaccine is highly cost-effective in comparison to the quadrivalent vaccine (ICER 4483€/QALY) and cost-saving compared to HPV2. This is a novel result in the Italian setting, as previous comparisons did not include the nine-valent vaccine [9, 22].

The results also suggest that universal vaccination with the nine-valent vaccine could be a cost-effective if compared to girls-only vaccination with the quadrivalent vaccine. The benefits of introducing male vaccination have previously been demonstrated with the quadrivalent vaccine in Italy with the BEST II study [19, 22]. A universal vaccination strategy with the nine-valent vaccine was still cost-effective against the quadrivalent vaccine universal (ICER 10,463€/QALY) and the quadrivalent vaccine girls-only (ICER 13,541€/QALY). Sensitivity analyses showed that results were mostly robust. The ICER was very sensitive to discount rates and the duration of protection. Using a higher discount rate or a reduced duration of protection significantly increased the ICER while no discount or a 1.5% discount rate only for outcomes substantially decreased the ICER. A lower coverage rate in both boys and girls or a lower adherence in males resulted in an ICER almost unchanged. This sensitivity to discount rates was expected since benefits of HPV vaccination occur several years after vaccination. The assumption of lifetime vaccine efficacy used in the base case was based on immunogenicity results and long-term protection of the vaccine available for the quadrivalent vaccine as well as models of long-term immune response [57, 58]. Thus, it is expected that the duration of protection against HPV genotypes 6/11/16/18/31/33/45/52/58 will be the same as the one for the quadrivalent vaccine since the two vaccines have structural similarities.

Showing potential cost-saving results is rarely seen for a new intervention, but our conclusion is in line with results obtained in the US and in Canada. In the US, three studies presented during the ACIP meeting showed that a universal vaccination programme with the nine-valent vaccine was likely to be cost effective and even cost saving compared to the current universal vaccination programme with the quadrivalent vaccine [29–31]. Brisson et al. and Chesson et al. found the nine-valent vaccine cost saving with respect to the quadrivalent vaccine if the price premium is below $13 per dose [33, 34]. In Canada, Drolet et al. showed the cost-effectiveness of the nine-valent vaccine if compared to the quadrivalent vaccine with a price premium of $11 [32].

Since in Italy vaccination coverage rates of girls are lower than expected, passing to universal vaccination in Italian regions where only girls are vaccinated with the bivalent or quadrivalent vaccine would have several benefits. First, it would protect females and males against HPV-related diseases and significantly reduce the remaining burden in both genders. Immunization of boys would indirectly protect girls from cancer and also be directly effective in the prevention of HPV-related diseases in men such as anal cancer [59, 60]. Moreover adding HPV vaccination of boys would decrease gender inequalities by protecting men exposed to male partners or unvaccinated females. Finally, it would allow HPV vaccination to become a standard vaccination in pre-adolescents [59].

Moreover, in the study only precancerous cervical lesions and cervical cancers were considered. Therefore, the impact on genital warts and anal cancers were not taken into account which nevertheless represents a substantial burden of HPV-related diseases. Indeed, in Italy, an economic study reported that the costs associated with genital warts in men and women corresponded to almost one quarter of the total costs associated with HPV 6, 11, 16, 18 [15, 59].

It is important to mention that our results may underestimate the additional benefits of the nine-valent vaccine vaccination on CIN. In the study from Hartwig et al. results showed that the HPV6/11/16/18 are responsible for about 24% of CIN1, 45% of CIN2+, 51% of vaginal cancer and 14% of vulvar cancer whereas HPV6/11/16/18/31/33/45/52/58 targeted by the nine-valent vaccine account for 48, 82, 61 and 16% of CIN1, CIN2+, vaginal cancer and vulvar cancer respectively [13]. It means that the 9-valent HPV infections were responsible for twice as much of CIN1 and 1.8 times more for CIN2+ compared the 4-valent HPV whereas the results obtained from the calibrated model indicated that 9-valent HPV accounts for 1.3 times more for CIN1 and 1.2 times more for CIN2+ than 4-valent HPV, minimizing greatly the benefit of the nine-valent vaccine on CIN compared to the quadrivalent vaccine. Moreover, the 9-valent HPV vaccine with the five additional HPV types is likely to protect more against vaginal and vulvar cancers compared to the quadrivalent vaccine and HPV2 [13]. Therefore, the nine-valent vaccine benefits on CIN, vaginal and vulvar cancers are probably underestimated in the present analysis.

Second, the model did not consider neonatal morbidity and mortality due to cervical lesions. Indeed, women treated for CIN with excisional treatments are at increased risk of preterm delivery and low birth weight [61, 62]. By decreasing the number of women treated for CIN, the nine-valent vaccine vaccination could reduce preterm birth number and decrease neonatal morbidity and mortality. Finally, the indirect costs related to productivity losses were not considered in this study. However HPV-related cancers affect women and men at work and their productivity. A study from Lerner et al. (2010) assessed the work performance and productivity impact of HPV in the US, and results showed that employed women with HPV-related cervical lesions had significantly more at-work limitations, higher absence rates and significantly more productivity losses because of absences compared with healthy controls [63].

The duration of protection of the nine-valent vaccine, as well as the quadrivalent vaccine, is not known. In the base case scenario, a lifelong protection was assumed, in accordance with the literature [22]. In order to check the robustness of the results, in the sensitivity analysis the duration of protection was set as short as 20 years. Results showed that the duration of protection had a great impact on the cost-effectiveness. However the ICERs remained below the range of €25,000–€40,000 per QALY [considered as cost-effective in Italy by the AIES (Italian Association of Health Economics)] for all scenarios except for the nine-valent vaccine universal versus the quadrivalent vaccine universal for a 10-year protection duration, indicating that vaccination of boys and girls aged 12 years remained a cost-effective strategy [49].

The major strength of this analysis is that the adaptation of this model originally designed for the US, was achieved through collection and selection of the appropriate data to reflect the Italian current epidemiological, medical and economical context. After the calibration process, the model was able to reproduce closely the observed incidence and mortality of HPV-related diseases in Italy.

This study has some limitations. One limitation of this analysis is that the model involved numerous parameters and not all relevant parameters could be found from Italian-specific studies, which may limit the validity of the results. However, non-Italian specific values have been validated by experts and usually refer to population-independent parameters. In order to simplify the calculations in the model, identical attribution to each of the five additional 9vHPV types, 31, 33, 45, 52, 58 was included. Finally, probabilistic sensitivity analysis was not performed because the interface of the model does not allow to quickly modify and evaluate several scenarios. However, the deterministic sensitivity analyses conducted to address the uncertainty showed that the conclusions of our analysis are robust.

Regarding screening practices, the pap smear test was used in the model and may not well represent the screening programme in Italy as this type of screening is being replaced by HPV-DNA testing in the country, and it could affect the cost effectiveness results. HPV-DNA test was not included in the analysis because Italian-specific data on the implementation of the HPV-DNA test are scarce, and the model does not allow for a flexibility in the use of mixed screening strategies.

## Conclusion

This analysis showed the additional benefits of the new technology for the Italian population. With a vaccination coverage rate of about 70% with the nine-valent vaccine, future population will be less exposed to high-risk HPV type and thus the burden of HPV-related diseases in Italy could consequently be reduced. The vaccination of girls only and universal vaccination with the nine-valent vaccine are cost-effective strategies compared with the vaccination of girls with the bivalent or the quadrivalent vaccine.

## Additional file

**Additional file 1.** Parameters of the epidemiological model.

## Abbreviations

ACIP: advisory committee on immunization practices
CIN: cervical intraepithelial neoplasia
HPV: human papilloma virus
ICER: incremental cost-effectiveness ratio
ICO: Institut Catala’ d’Oncologia
QALY: quality adjusted life year
RRP: recurrent respiratory papillomatosis
SIRS: susceptible-infected-recovered-susceptible
UK: United Kingdom
US: United states
VaIN: vaginal intraepithelial neoplasia
VIN: vulvar intraepithelial neoplasia
WHO: World Health Organization

## Footnotes

1 As of July 6th 2015 http://www.cdc.gov/vaccines/programs/vfc/awardees/vaccine-management/price-list/: USD = 0.906249 EUR.
