# Supplementary material for: Cost-effectiveness analysis of the nine-valent HPV vaccine in Italy
Source: Cost Eff Resour Alloc. 2017 Jul 11;15:11. doi: 10.1186/s12962-017-0073-8 (PMC5504713; doi:10.1186/s12962-017-0073-8)
Supplement: Supplementary file 1 — Additional file 1. Parameters of the epidemiological model. [file 12962_2017_73_MOESM1_ESM.docx]

**Supplementary material**

Epidemiologic model parameters

The main epidemiological model inputs are summarised in Table 5 and displayed in detail in the following tables.

Table 5: Summary table of the references used for the epidemiological parameters

| Parameter | | References |
| --- | --- | --- |
| *Demographics* | |  |
|  | Annual all-cause mortality rate | ISTAT [10] |
|  | Female and male population | ISTAT [10] |
|  | Female and male population >12yo | ISTAT [10] |
| *Sexual behavior* | |  |
|  | Percent of population in low/medium/high sexual activity category | NATSAL III* |
|  | Mean number of sexual partners by activity category | NATSAL III* |
|  | Mean number of sexual partners by age group | NATSAL III* |
|  | Sexual mixing among activity categories | NATSAL III* |
|  | Sexual mixing among age groups | Elbasha et al. (2010)  [19, 20] |
| *Disease and treatment patterns* | |  |
|  | Female population receiving hysterectomy each year | Mennini at al. (2009) [9] |
|  | Age- and stage- specific mortality rates | Haeussler et al. (2015), ITACAN, EUROCARE [22, 37, 64] |
| *Screening* | |  |
|  | Percent of females receiving a follow-up screening test | Haeussler et al. (2015), [22] |
|  | Percent of females screened every 3 years | Osservatorio Nazionale Screening [38] |
|  | Age-specific percent of females screened in the past year | Haeussler et al. (2015), Osservatorio Nazionale Screening [22, 38] |
|  | Diagnostic performance of PAP test and colposcopy | Mennini et al. (2009) [9] |
| *Natural history of disease* | |  |
|  | Probability of transmitting genital, anal, penile, and head and neck HPV infection per sexual partnership, by sex and HPV genotype | Hernandez et al. (2008) [65] |
|  | Recurrence rate of treated CIN, by stage |  |
|  | Rate of cancer progression, by stage | Myers et al. (2000) Sanders (2003) [66, 67] |
|  | Fraction of persistent cervical HPV infections, by type 16 or 18 | Elbasha et al. (2010) [19] |
|  | Clearance rate of cervical HPV infections, by type 16 or 18 | Insinga et al. (2007) [68] |
|  | Fraction of people who seroconvert following a cervical HPV infection, by type 16 or 18 | Ho et al. (2004), Onda et al. (2003) [69, 70] |
|  | Degree of protection against cervical HPV infections provided by natural immunity following seroconversion, by type 16 or 18 | Elbasha et al. (2010) [19] |
|  | Fraction of females transiently infected who progress to CIN over the course of one year, by type 16 or 18 | Insinga et al. (2007) [68] |
|  | * Data were not obtained from the article but from email communication with the NATSAL team | |

Table 6: Summary table on sexual behaviour inputs

| Definition of sexual activity categories | | | | |  |  | Mean number of sexual partners per year | | |
| --- | --- | --- | --- | --- | --- | --- | --- | --- | --- |
| Low | | mean number of sexual partners/year: ≤ 1 | | | | | Age Group | Male | Female |
| Medium | | mean number of sexual partners/year: 2-4 | | | | | 13-14 | 0.0001 | 0.0001 |
| High | | mean number of sexual partners/year: 5+ | | | | | 15-29 | 1.7 | 1.4 |
|  | | | | | |  | 30-34 | 2 | 1.6 |
| Size of categories and mean number of partners | | | | | | | 35-44 | 1.7 | 1.3 |
|  | | Percent of population | | | Mean number of partners | | 45-49 | 1.5 | 1.2 |
|  | | Male | | Female | Males | Females | 50-54 | 1.5 | 1 |
| Low | | 85.10% | | 90.70% | 0.79 | 0.75 | 55-59 | 1.1 | 1.5 |
| Medium | | 11.90% | | 7.60% | 2.54 | 2.52 | 60-64 | 1.1 | 1 |
| High | 3.00% | | 1.70% | | 9.8 | 9.66 | 65-69 | 1.1 | 0.9 |
|  | | | | | |  | 70-74 | 1 | 0.7 |
| Sexual mixing | | | | | |  | 75-79 | 0.9 | 0.6 |
| Among members of different age groups | | | | | | 0.4 | 80-84 | 0.8 | 0.5 |
| Among members of different sexual activity groups | | | | | | 0.5 | 85+ | 0.5 | 0.3 |
| * Debut and cessation referes to the period in which sexual activity occurs. | | | | | | | | | |

Table 7: Summary table on the natural history of disease

| Probability of transmitting genital HPV infection [62] | | | | | |
| --- | --- | --- | --- | --- | --- |
| Transmission | HPV 16 | HPV 18 | HPV 6 | HPV 11 | HPV 31, 33, 45, 52 or 58 |
| To males | 0.1109 | 0.1109 | 0.2577 | 0.2577 | 0.076 |
| To females | 0.1109 | 0.1109 | 0.2577 | 0.2577 | 0.076 |
| Stage | Recurrence rate | Reference | Cancer progression |  | Reference |
| CIN 1 | 0.05 | Assumption | Direction | Rate |  |
| CIN 2 | 0.05 | Assumption | local->regional | 0.1 | [66, 67] |
| CIN 3 | 0.05 | Assumption | regional->distant | 0.3 | [66, 67] |
| Parameters (References) | | | | HPV 16 | HPV 18 |
| Fraction of persistent cervical HPV infections (Elbasha)[20] | | | | 0.25 | 0.075 |
| *Clearance rate of cervical HPV infections (Insinga[68])* | | | |  |  |
| Male | | | | 0.3955 | 0.37755 |
| Female | | | | 0.354 | 0.348 |
| *Fraction of people who seroconvert following a cervical HPV infection (Ho [69] Onda [70]])* | | | |  |  |
| Male | | | | 0.6 | 0.6 |
| Female | | | | 0.6 | 0.6 |
| *Degree of protection against cervical HPV infections provided by natural immunity following seroconversion (Elbasha [20])* | | | |  |  |
| Male | | | | 0.5 | 0.5 |
| Female | | | | 0.8 | 0.8 |
| *Fraction of females transiently infected with HPV16 who progress to CIN over the course of one year (Insinga [68])* | | | |  |  |
| CIN 1 | | | | 0.105 | 0.068 |
| CIN 2 | | | | 0.045 | 0.055 |
| CIN 3 | | | | 0.024 | 0.009 |
| *Probability of transmitting anal HPV infection (Calibration)* | | | |  |  |
| To males | | | | 0.16 | 0.16 |
| To females | | | | 0.173 | 0.173 |
| *Probability of transmitting penile HPV infection (Calibration)* | | | |  |  |
| To males | | | | 0.123 | 0.123 |
| To females | | | | 0.123 | 0.123 |
| *Probability of transmitting head and neck HPV infection (Calibration)* | | | |  |  |
| To males | | | | 0.14118 | 0.13228 |
| To females | | | | 0.14118 | 0.13228 |

Table 8: Summary table on cancer mortality

| Cancer type | Age group | Annual probability of death | | |
| --- | --- | --- | --- | --- |
|  | (years) | Local Cervical Cancer | Regional Cervical Cancer | Distant Cervical Cancer |
| Cervical cancer | 15-44 | 0.04 | 0.1 | 0.11 |
|  | 45-54 | 0.06 | 0.15 | 0.17 |
|  | 55-64 | 0.07 | 0.19 | 0.21 |
|  | 65-74 | 0.12 | 0.3 | 0.32 |
|  | 75+ | 0.23 | 0.52 | 0.56 |
| Vaginal cancer | 15-44 | 0.06 | 0.16 | 0.4 |
|  | 45-54 | 0.07 | 0.18 | 0.43 |
|  | 55-64 | 0.08 | 0.21 | 0.48 |
|  | 65-74 | 0.11 | 0.27 | 0.6 |
|  | 75+ | 0.2 | 0.46 | 0.83 |
| Vulvar cancer | 15-44 | 0.06 | 0.1 | 0.17 |
|  | 45-54 | 0.07 | 0.11 | 0.19 |
|  | 55-64 | 0.08 | 0.12 | 0.22 |
|  | 65-74 | 0.11 | 0.17 | 0.29 |
|  | 75+ | 0.2 | 0.3 | 0.48 |
| Anal cancer (Females) | 15-44 | 0.08 | 0.09 | 0.1 |
|  | 45-54 | 0.08 | 0.09 | 0.1 |
|  | 55-64 | 0.08 | 0.1 | 0.11 |
|  | 65-74 | 0.1 | 0.13 | 0.14 |
|  | 75+ | 0.21 | 0.26 | 0.28 |
| Anal cancer (Males) | 15-44 | 0.08 | 0.1 | 0.11 |
|  | 45-54 | 0.09 | 0.11 | 0.13 |
|  | 55-64 | 0.1 | 0.12 | 0.13 |
|  | 65-74 | 0.12 | 0.15 | 0.16 |
|  | 75+ | 0.21 | 0.26 | 0.28 |
| Penile cancer | 15-44 | 0.04 | 0.07 | 0.16 |
|  | 45-54 | 0.05 | 0.07 | 0.17 |
|  | 55-64 | 0.04 | 0.07 | 0.16 |
|  | 65-74 | 0.09 | 0.13 | 0.3 |
|  | 75+ | 0.19 | 0.28 | 0.57 |
| Head & Neck cancer (Females) | 15-44 | 0.07 | 0.17 | 0.18 |
|  | 45-54 | 0.1 | 0.26 | 0.27 |
|  | 55-64 | 0.11 | 0.28 | 0.29 |
|  | 65-74 | 0.11 | 0.29 | 0.3 |
|  | 75+ | 0.23 | 0.52 | 0.54 |
| Head & Neck cancer (Males) | 15-44 | 0.11 | 0.27 | 0.28 |
|  | 45-54 | 0.14 | 0.35 | 0.37 |
|  | 55-64 | 0.15 | 0.38 | 0.39 |
|  | 65-74 | 0.19 | 0.46 | 0.47 |
|  | 75+ | 0.27 | 0.59 | 0.61 |

Table 9. Cervical cancer screening rate

| Age group | Percentage of females screened in the past year | Reference |
| --- | --- | --- |
| 0-10 | 0% | Assumption |
| 11-24 | 5% | [21] |
| 25-34 | 37% | [39] |
| 35-44 | 42% | [39] |
| 45-54 | 43% | [39] |
| 55-64 | 41% | [39] |
| 65-74 | 18% | [21] |
| >75 | 0% | Assumption |

**Additional references**

64. Airtum, *ITACAN database*.

65. Hernandez, B.Y., et al., *Transmission of human papillomavirus in heterosexual couples.* Emerg.Infect.Dis., 2008. **14**(6):

66. Myers, E.R., et al., *Mathematical model for the natural history of human papillomavirus infection and cervical carcinogenesis.* Am.J.Epidemiol., 2000. **151**(12): p. 1158-1171.

67. Sanders, G.D. and A.V. Taira, *Cost-effectiveness of a potential vaccine for human papillomavirus.* Emerg.Infect.Dis., 2003. **9**(1): p. 37-48.

68. Insinga, R.P., et al., *Progression and regression of incident cervical HPV 6, 11, 16 and 18 infections in young women.* Infect.Agent.Cancer, 2007. **2**: p. 15.

69. Ho, G.Y., et al., *Natural history of human papillomavirus type 16 virus-like particle antibodies in young women.* Cancer Epidemiol.Biomarkers Prev., 2004. **13**(1): p. 110-116.

70. Onda, T., et al., *Characterization of IgA response among women with incident HPV 16 infection.* Virology, 2003. **312**(1): p. 213-221.
